# Supplementary material for: Treatment provision for adults with ADHD during the COVID-19 pandemic: an exploratory study on patient and therapist experience with on-site sessions using face masks vs. telepsychiatric sessions
Source: BMC Psychiatry. 2021 May 5;21:237. doi: 10.1186/s12888-021-03236-9 (PMC8097668; doi:10.1186/s12888-021-03236-9)
Supplement: Supplementary file 1 — Additional file 1: Table A. Overview of self-developed questions used in the study (German original wording and English translation). Table B. German Original Wording and English Translations of the Quotes Cited in the Publication. Table C Hierarchical Regression Results for SEQ Depth. Table D. Hierarchical Regression Results for SEQ Smoothness. Table E. Hierarchical Regression Results for SEQ Positivity. Table F. Hierarchical Regression Results for CSQ. Table G. Hierarchical Regression Results for WAI Bond (Patient). Table H. Hierarchical Regression Results for WAI Tasks (Patient). Table I. Hierarchical Regression Results for WAI Goals (Patient). Table J. Hierarchical Regression Results for WAI Bond Therapist. Table K. Hierarchical Regression Results for WAI Tasks Therapist. Table L. Hierarchical Regression Results for WAI Goals Therapist. Table M. Sensitivity Analyses: Adjusted Standardised Regression Coefficients for Face-to-Face vs. Telephone Modality. [file 12888_2021_3236_MOESM1_ESM.docx]

**Treatment provision for adults with ADHD during the COVID-19 pandemic: An exploratory study on patient and therapist experience with on-site sessions using face masks vs. telepsychiatric sessions**

Helen Wyler^*1^, Michael Liebrenz^*1^, Vladeta Ajdacic-Gross^2^, Erich Seifritz^2^, Susan Young^3 4^, Pascal Burger^2^, & Anna Buadze^2^

^1^ Department of Forensic Psychiatry, Institute of Forensic Medicine, University of Bern, Bern, Switzerland

^2^ Department of Psychiatry, Psychotherapy and Psychosomatics, Psychiatric Hospital, University of Zurich, Zurich, Switzerland

^3^ Psychology Services Limited, London, UK

^4^ Department of Psychology, Reykjavik University, Iceland

*These authors are co-first authors on this work.

**Author Note**

We have no conflicts of interest to disclose.

Correspondence concerning this article should be addressed to Helen Wyler, Department of Forensic Psychiatry, Institute of Forensic Medicine, University of Bern, Falkenplatz 18, 3012 Bern, Switzerland. Email: helen.wyler@fpd.unibe.ch

**Supplementary File 1**

**Table A**

*Overview of Self-Developed Questions Used in the Study (German Original Wording and English Translation)*

| **Item** | **German original** | **English translation** |
| --- | --- | --- |
| C | Anleitung: Bitte beantworten Sie die vier folgenden Fragen dazu, wie Sie die COVID-19 Pandemie erleben.  Antwortoptionen C1: ja/nein  Antwortoptionen C2-C4:   - *überhaupt nicht (1)* - *(2)* - *(3)* - *(4)* - *sehr stark (5)* | Instruction: Please answer the following four questions about how you are experiencing the COVID-19 pandemic.  Response options C1: yes/no  Response options C2-C4:   - *not at all (1)* - *(2)* - *(3)* - *(4)* - *very much (5)* |
| C1 | Gehören Sie zu jenen Personen, die ein erhöhtes Risiko für einen schwereren Verlauf einer COVID-19-Erkrankung haben (dies sind Personen ab 65 Jahren oder Personen mit mindestens einer der folgenden Vorerkrankungen: Bluthochdruck, chronische Atemwegserkrankungen, Diabetes, Erkrankungen und Therapien, die das Immunsystem schwächen, Herz-Kreislauf-Erkrankungen, Krebs)? | Do you belong to the group of people who are at a higher risk of developing more severe illness from COVID-19 (these are people aged 65 and over or people with at least one of the following pre-existing conditions: High blood pressure, chronic respiratory disease, diabetes, diseases and therapies that weaken the immune system, cardiovascular disease, cancer)? |
| C2 | **In welchem Ausmass hat die COVID-19 Pandemie Ihren Alltag verändert?** | To what extent has the COVID-19 pandemic affected your everyday life? |
| C3 | **Inwiefern fühlen Sie sich wegen der COVID-19 Pandemie gestresst?** | To what extent do you feel distressed because of the COVID-19 pandemic? |
| C4 | **Inwiefern löst die COVID-19 Pandemie bei Ihnen Angst aus?** | To what extent does the COVID-19 pandemic trigger fear? |
| OP1 | Gab es etwas an der heutigen Sitzungsform, das Ihnen besonders gefallen hat?  *Offene Frage* | Was there anything you particularly liked about the way today’s session took place?  *Open-ended question* |
| OP2 | Gab es etwas an der heutigen Sitzungsform, das Sie gestört hat?  *Offene Frage* | Was there anything you felt uncomfortable with or disliked about the way today’s session took place?  *Open-ended question* |
| OT1 | Wie bewerten Sie die aktuell durchgeführte Therapieform im Vergleich zu der normalerweise durchgeführten Therapieform bei diesem Patienten/dieser Patientin?  *Offene Frage* | For this patient, how do you evaluate the way the therapy sessions are currently conducted compared to the way they normally take place?  *Open-ended question* |
| F1 | Wie gefällt Ihnen die Art und Weise, wie die heutige Sitzung stattgefunden hat, im Vergleich zu den «normalen» Therapiesitzungen vor dem Pandemieausbruch (persönlich vor Ort und ohne Schutzmassnahmen)?   - *Die «normalen» Therapiesitzungen waren besser* - *Die Sitzungen so wie heute sind besser* - *Für mich macht es keinen Unterschied* | How do you like the way today’s session took place compared to the “normal” therapy sessions before the pandemic outbreak (in person, on site and without protective measures)?   - *The “normal” therapy sessions were better* - *The sessions as the one today are better* - *It makes no difference to me* |
| F2 | Bitte ordnen Sie die folgenden Sitzungsmöglichkeiten so an, dass ganz oben jene Möglichkeit ist, die Sie sich am meisten wünschen würden (=1) und ganz unten jene Möglichkeit ist, die Sie sich am wenigsten wünschen würden (=5).   - *Telefon* - *Videotelefonie (z.B. Jitsi oder Skype)* - *Persönlich vor Ort, als Schutzmassnahme werden die gängigen Hygieneregeln beachtet (kein Händeschütteln, mindestens 2 Meter Abstand)* - *Persönlich vor Ort, Therapeut/in trägt als Schutzmassnahme eine Schutzmaske* - *Persönlich vor Ort, als Schutzmassnahme hat es eine Trennscheibe zwischen mir und dem/der Therapeuten/Therapeutin* | Please arrange the following options in a way that the most preferred is on top (=1) and the least preferred is at the bottom (=5).   - *Telephone* - *Videoconferencing (e.g. Jitsi or Skype)* - *In person and on site, as protective measures the usual hygiene rules are observed (no handshaking, at least 2 meters distance)* - *In person and on site, therapist wears a face mask as a protective measure* - *In person and on site, as a protective measure a plastic divider is situated between me and the therapist* |

*Note*: The SEQ, CSQ, WAI, and GAF, which were also used in this study, are pre-existing questionnaires. The relevant references can be found in the main manuscript.

**Table B**

*German Original Wording and English Translations of the Quotes Cited in the Publication*

| **Case** | **German original** | **English translation** |
| --- | --- | --- |
| Patient 30 | die Zeit und therapeutische Diskussion, die wir hatten | the time and therapeutic discussion we had |
| Patient 32 | Was meinen Therapeuten an sich betrifft, (…) ich bin sehr zufrieden. Jedoch ist das Tragen einer Maske ein wenig störend, da man die Gesichtsausdrücke nicht mehr richtig sehen kann. Ich verstehe aber natürlich die Notwendigkeit dieser Massnahme. | As for my therapist per se, (…), I am very satisfied. However, wearing a mask was a bit annoying because you can no longer see the facial expressions properly. But of course I understand the necessity of this measure. |
| Patient 25 | Es ist unkompliziert. Wenn der Arzt Verspätung hat geht keine Zeit verloren, da ich in der Zwischenzeit etwas sinnvolles machen kann, und nicht im Wartezimmer nichts tue. | It is straightforward. If the doctor is running late, no time is lost because I can do something useful in the meantime rather than doing nothing in the waiting room. |
| Patient 24 | (…) Gegenseitige Präsenz war tiefer als bei physischem Treffen. Dies kann auf meiner Seite daran liegen, dass durch den fehlende Weg zur Therapie der Bruch von Home Office zur Therapiestunde zu aprupt war. | (…) Mutual presence was deeper at physical meetings. On my part, this may be due to the fact that, because there was no travelling to the therapy session, the change from home office to therapy session was too abrupt. |
| Patient 21 | Das die Sitzung nur im Gespräch ohne Blick und Mimik statt findet, auch Vorteile, denn so bin ich mehr auf den Inhalt fokussiert und nicht über visuelle Reize oder dem Verhalten meines Therapeuten abgelenkt oder beeinflusst. | The fact that the session is a conversation only without look and facial expressions also has advantages, because this way I am more focused on the content and not distracted or influenced by visual stimuli or by my therapist’s behaviour. |
| Patient 28 | (…) Vieles was ich sonst visuell abschätzen kann, fehlte mir. zB. Ob meine Frage verstanden wurde, konnte ich an der Mimik nicht ablesen. Dies wurde erst bei der Antwort klar. (…) | (…) A lot of things that I can normally assess visually were missing. E.g., I couldn’t tell from the facial expressions whether my question had been understood. This only became clear with the [therapist’s] response. (…) |
| Therapist B, Patient 39 | Hat anfängliche Schwierigkeiten sich mit der Schutzmaske zu arrangieren, (…) fragt immer wieder nach und meint mich akustisch nicht verstanden zu haben. Dies unterbricht den Gesprächsfluss | Has initial difficulties coming to terms with the face mask, (…) keeps asking questions and thinks s(he) has not understood me acoustically. This interrupts the flow of conversation. |
| Therapist A, Patient 10 | Einschränkung durch Maske, wegen deutlicher Reduktion der mimischen Antwort und leichte Verständlichkeitsprobleme - allerdings bei jungem, (…) reflektiertem Patienten ausreichend gut durchführbare Sitzung. | Barrier due to mask because of significant reduction of mimic response and slight problems with comprehensibility – however, with a young, (…) reflective patient the session was sufficiently feasible. |
| Therapist B, Patient 35 | Üblicherweise meidet dieser Patient den Blickkontakt. Unter diesen Umständen hielt er diesen aber auffällig häufig aufrecht (…) | usually, this patient avoids eye contact. Under these circumstances, however, s(he) maintained eye contact remarkably well (…) |
| Therapist B, Patient 40 | Es entsteht der Eindruck, dass durch die verdeckte Mundpartie bei mir, der Patient besser aufpasst und nicht wie üblich abschweift. | The impression is given that the covering of the region of the mouth made the patient pay more attention and not drifting off as [much as] usual. |
| Therapist A, Patient 24 | eingeschränkt aufgrund der nicht komplett einsehbaren körperlichen Antwort und der massiv erschwerten konkreten Arbeitsmöglichkeit mit dem Patienten (…) To-Do-Liste generieren (…) | limited due to the physical response not being completely visible and the considerable difficulties in completing specific tasks with the patient, (…) e.g. generating a to-do list (…) |
| Therapist A, Patient 28 | Fehlen der emotionalen Antwort bzw. von deren Äquivalenten in Mimik und Gestik. Patient gut bekannt, dennoch deutliche Einschränkung dadurch | Absence of emotional response or its equivalents in facial expressions and gestures. Patient is well known, but limitations were still significant because of it [the lack of visual information] |
| Therapist B, Patient 51 | Da kürzlich ein Elternteil verstorben ist, ist es schwierig alles nur per Telefon zu besprechen. Es wäre für den Patienten besser auch zur Entlastung die Wohnung zu verlassen | As one parent recently passed away, it is difficult to discuss everything only over the phone. It would be better for the patient to leave his/her home to ease the burden [of the loss] as well |

*Note: To protect the patient’s and therapists’ identities, all German original quotes presented were changed to the generic masculine.*

**Table C**

*Hierarchical Regression Results for SEQ Depth*

| Variable | *B* | 95% CI for *B* | | *SE B* | β | *R^2^* | Δ*R^2^* |
| --- | --- | --- | --- | --- | --- | --- | --- |
|  |  | *LL* | *UL* |  |  |  |  |
| Step 1 |  |  |  |  |  | .01 | .00 |
| Constant | 5.25^***^ | 4.70 | 5.80 | 0.28 |  |  |  |
| Therapist | -0.18 | -0.68 | 0.32 | 0.25 | -.10 |  |  |
| No of sessions | 0.01 | -0.16 | 0.18 | 0.09 | .02 |  |  |
| Step 2 |  |  |  |  |  | .18^*^ | .13 |
| Constant | 5.25^***^ | 4.72 | 5.77 | 0.26 |  |  |  |
| Therapist | -0.10 | -0.57 | 0.36 | 0.23 | -.06 |  |  |
| No of sessions | 0.18 | -0.01 | 0.37 | 0.09 | .28 |  |  |
| Modality | -0.87^**^ | -1.42 | -0.32 | 0.28 | -.46 |  |  |

*Note*: Modality: 0 = face-to-face, 1 = telepsychiatry. ^*^*p* < .05. ^**^*p* < .01. ^***^*p* < .001.

**Table D**

*Hierarchical Regression Results for SEQ Smoothness*

| Variable | *B* | 95% CI for *B* | | *SE B* | β | *R^2^* | Δ*R^2^* |
| --- | --- | --- | --- | --- | --- | --- | --- |
|  |  | *LL* | *UL* |  |  |  |  |
| Step 1 |  |  |  |  |  | .09 | .06 |
| Constant | 6.08^***^ | 5.44 | 6.71 | 0.32 |  |  |  |
| Therapist | -0.65^*^ | -1.21 | -0.09 | 0.28 | -.30 |  |  |
| No of sessions | -0.10 | -0.29 | 0.09 | 0.10 | -.14 |  |  |
| Step 2 |  |  |  |  |  | .10 | .05 |
| Constant | 6.08^***^ | 5.44 | 6.71 | 0.32 |  |  |  |
| Therapist | -0.63^*^ | -1.19 | -0.06 | 0.28 | -.29 |  |  |
| No of sessions | -0.06 | -0.29 | 0.17 | 0.11 | -.08 |  |  |
| Modality | -0.22 | -0.89 | 0.45 | 0.33 | -.10 |  |  |

*Note*: Modality: 0 = face-to-face, 1 = telepsychiatry. ^*^*p* < .05. ^**^*p* < .01. ^***^*p* < .001.

**Table E**

*Hierarchical Regression Results for SEQ Positivity*

| Variable | *B* | 95% CI for *B* | | *SE B* | β | *R^2^* | Δ*R^2^* |
| --- | --- | --- | --- | --- | --- | --- | --- |
|  |  | *LL* | *UL* |  |  |  |  |
| Step 1 |  |  |  |  |  | .00 | .00 |
| Constant | 5.49^***^ | 4.86 | 6.13 | 0.32 |  |  |  |
| Therapist | -0.12 | -0.69 | 0.44 | 0.28 | -.06 |  |  |
| No of sessions | -0.02 | -0.21 | 0.17 | 0.10 | -.03 |  |  |
| Step 2 |  |  |  |  |  | .01 | .00 |
| Constant | 5.49^***^ | 4.85 | 6.13 | 0.32 |  |  |  |
| Therapist | -0.14 | -0.71 | 0.44 | 0.29 | -.06 |  |  |
| No of sessions | -0.04 | -0.27 | 0.19 | 0.12 | -.06 |  |  |
| Modality | 0.12 | -0.56 | 0.79 | 0.34 | -.06 |  |  |

*Note*: Modality: 0 = face-to-face, 1 = telepsychiatry. ^*^*p* < .05. ^**^*p* < .01. ^***^*p* < .001.

**Table F**

*Hierarchical Regression Results for CSQ*

| Variable | *B* | 95% CI for *B* | | *SE B* | β | *R^2^* | Δ*R^2^* |
| --- | --- | --- | --- | --- | --- | --- | --- |
|  |  | *LL* | *UL* |  |  |  |  |
| Step 1 |  |  |  |  |  | .01 | .00 |
| Constant | 3.70^***^ | 3.51 | 3.88 | 0.09 |  |  |  |
| Therapist | -0.03 | -0.20 | 0.13 | 0.08 | -.05 |  |  |
| No of sessions | 0.01 | -0.04 | 0.07 | 0.03 | .07 |  |  |
| Step 2 |  |  |  |  |  | .05 | .00 |
| Constant | 3.79^***^ | 3.51 | 3.88 | 0.09 |  |  |  |
| Therapist | -0.02 | -0.19 | 0.15 | 0.08 | -.03 |  |  |
| No of sessions | 0.04 | -0.03 | 0.11 | 0.03 | .20 |  |  |
| Modality | -0.14 | -0.34 | 0.05 | 0.10 | -.23 |  |  |

*Note*: Modality: 0 = face-to-face, 1 = telepsychiatry. ^*^*p* < .05. ^**^*p* < .01. ^***^*p* < .001.

**Table G**

*Hierarchical Regression Results for WAI Bond Patient*

| Variable | *B* | 95% CI for *B* | | *SE B* | β | *R^2^* | Δ*R^2^* |
| --- | --- | --- | --- | --- | --- | --- | --- |
|  |  | *LL* | *UL* |  |  |  |  |
| Step 1 |  |  |  |  |  | .00 | .00 |
| Constant | 4.33^***^ | 3.97 | 4.69 | 0.18 |  |  |  |
| Therapist | 0.02 | -0.30 | 0.34 | 0.16 | .01 |  |  |
| No of sessions | 0.00 | -0.11 | 0.11 | 0.05 | .00 |  |  |
| Step 2 |  |  |  |  |  | .02 | .00 |
| Constant | 4.34^***^ | 3.97 | 4.70 | 0.18 |  |  |  |
| Therapist | 0.03 | -0.29 | 0.36 | 0.16 | .03 |  |  |
| No of sessions | 0.03 | -0.10 | 0.16 | 0.07 | .09 |  |  |
| Modality | -0.19 | -0.57 | 0.19 | 0.19 | -.16 |  |  |

*Note*: Modality: 0 = face-to-face, 1 = telepsychiatry. ^*^*p* < .05. ^**^*p* < .01. ^***^*p* < .001.

**Table H**

*Hierarchical Regression Results for WAI Tasks Patient*

| Variable | *B* | 95% CI for *B* | | *SE B* | β | *R^2^* | Δ*R^2^* |
| --- | --- | --- | --- | --- | --- | --- | --- |
|  |  | *LL* | *UL* |  |  |  |  |
| Step 1 |  |  |  |  |  | .01 | .00 |
| Constant | 3.93^***^ | 3.57 | 4.29 | 0.18 |  |  |  |
| Therapist | -0.12 | -0.45 | 0.20 | 0.16 | -.10 |  |  |
| No of sessions | 0.00 | -0.11 | 0.11 | 0.05 | .01 |  |  |
| Step 2 |  |  |  |  |  | .06 | .01 |
| Constant | 3.94^***^ | 3.58 | 4.29 | 0.18 |  |  |  |
| Therapist | -0.10 | -0.41 | 0.22 | 0.16 | -.08 |  |  |
| No of sessions | 0.06 | -0.06 | 0.19 | 0.06 | .16 |  |  |
| Modality | -0.33 | -0.70 | 0.05 | 0.19 | -.27 |  |  |

*Note*: Modality: 0 = face-to-face, 1 = telepsychiatry. ^*^*p* < .05. ^**^*p* < .01. ^***^*p* < .001.

**Table I**

*Hierarchical Regression Results for WAI Goals Patient*

| Variable | *B* | 95% CI for *B* | | *SE B* | β | *R^2^* | Δ*R^2^* |
| --- | --- | --- | --- | --- | --- | --- | --- |
|  |  | *LL* | *UL* |  |  |  |  |
| Step 1 |  |  |  |  |  | .00 | .00 |
| Constant | 3.99^***^ | 3.61 | 4.37 | 0.19 |  |  |  |
| Therapist | 0.05 | -0.29 | 0.38 | 0.17 | .04 |  |  |
| No of sessions | -0.02 | -0.13 | 0.10 | 0.06 | -.04 |  |  |
| Step 2 |  |  |  |  |  | .01 | .00 |
| Constant | 3.99^***^ | 3.61 | 4.37 | 0.19 |  |  |  |
| Therapist | 0.04 | -0.30 | 0.38 | 0.17 | .03 |  |  |
| No of sessions | -0.04 | -0.17 | 0.10 | 0.07 | -.08 |  |  |
| Modality | 0.10 | -0.30 | 0.50 | 0.20 | .08 |  |  |

*Note*: Modality: 0 = face-to-face, 1 = telepsychiatry. ^*^*p* < .05. ^**^*p* < .01. ^***^*p* < .001.

**Table J**

*Hierarchical Regression Results for WAI Bond Therapist*

| Variable | *B* | 95% CI for *B* | | *SE B* | β | *R^2^* | Δ*R^2^* |
| --- | --- | --- | --- | --- | --- | --- | --- |
|  |  | *LL* | *UL* |  |  |  |  |
| Step 1 |  |  |  |  |  | .11^*^ | .08 |
| Constant | 3.85^***^ | 3.57 | 4.13 | 0.14 |  |  |  |
| Therapist | 0.29^*^ | 0.04 | 0.54 | 0.12 | .30 |  |  |
| No of sessions | 0.06 | -0.02 | 0.15 | 0.04 | .19 |  |  |
| Step 2 |  |  |  |  |  | .13^*^ | .09 |
| Constant | 3.85^***^ | 3.58 | 4.13 | 0.14 |  |  |  |
| Therapist | 0.31^*^ | 0.06 | 0.55 | 0.12 | .32 |  |  |
| No of sessions | 0.10 | 0.00 | 0.20 | 0.05 | .30 |  |  |
| Modality | -0.18 | -0.47 | 0.11 | 0.14 | -.19 |  |  |

*Note*: Modality: 0 = face-to-face, 1 = telepsychiatry. ^*^*p* < .05. ^**^*p* < .01. ^***^*p* < .001. *F* change was not significant for Step 2, which means that the inclusion of condition does not statistically significantly improve prediction of the outcome variable. As the outcome is based on the ratings from two therapists, the regressions were also run for each therapist individually, in which case *therapist* was removed as a predictor in Step 1. *Modality* was not a statistically significant predictor in either of the two hierarchical regressions.

**Table K**

*Hierarchical Regression Results for WAI Tasks Therapist*

| Variable | *B* | 95% CI for *B* | | *SE B* | β | *R^2^* | Δ*R^2^* |
| --- | --- | --- | --- | --- | --- | --- | --- |
|  |  | *LL* | *UL* |  |  |  |  |
| Step 1 |  |  |  |  |  | .10 | .06 |
| Constant | 3.48^***^ | 3.21 | 3.74 | 0.13 |  |  |  |
| Therapist | 0.25^*^ | 0.02 | 0.49 | 0.12 | .28 |  |  |
| No of sessions | 0.06 | -0.02 | 0.14 | 0.04 | .19 |  |  |
| Step 2 |  |  |  |  |  | .12 | .07 |
| Constant | 3.48^***^ | 3.22 | 3.74 | 0.13 |  |  |  |
| Therapist | 0.27^*^ | 0.03 | 0.50 | 0.12 | .29 |  |  |
| No of sessions | 0.09 | 0.00 | 0.18 | 0.05 | .29 |  |  |
| Modality | -0.17 | -0.44 | 0.11 | 0.14 | -.19 |  |  |

*Note*: Modality: 0 = face-to-face, 1 = telepsychiatry. ^*^*p* < .05. ^**^*p* < .01. ^***^*p* < .001. As the outcome is based on the ratings from two therapists, the regressions were also run for each therapist individually, in which case *therapist* was removed as a predictor in Step 1. *Modality* was not a statistically significant predictor in either of the two hierarchical regressions.

**Table L**

*Hierarchical Regression Results for WAI Goals Therapist*

| Variable | *B* | 95% CI for *B* | | *SE B* | β | *R^2^* | Δ*R^2^* |
| --- | --- | --- | --- | --- | --- | --- | --- |
|  |  | *LL* | *UL* |  |  |  |  |
| Step 1 |  |  |  |  |  | .05 | .02 |
| Constant | 3.46^***^ | 3.20 | 3.72 | 0.13 |  |  |  |
| Therapist | 0.19 | -0.04 | 0.43 | 0.12 | .22 |  |  |
| No of sessions | 0.04 | -0.04 | 0.12 | 0.04 | .12 |  |  |
| Step 2 |  |  |  |  |  | .05 | .00 |
| Constant | 3.46^***^ | 3.19 | 3.73 | 0.13 |  |  |  |
| Therapist | 0.20 | -0.04 | 0.43 | 0.12 | .22 |  |  |
| No of sessions | 0.04 | -0.05 | 0.14 | 0.05 | .14 |  |  |
| Modality | -0.03 | -0.31 | 0.25 | 0.14 | -.03 |  |  |

*Note*: Modality: 0 = face-to-face, 1 = telepsychiatry. ^*^*p* < .05. ^**^*p* < .01. ^***^*p* < .001. As the outcome is based on the ratings from two therapists, the regressions were also run for each therapist individually, in which case *therapist* was removed as a predictor in Step 1. *Modality* was not a statistically significant predictor in either of the two hierarchical regressions.

**Table M**

*Sensitivity Analyses: Adjusted Standardised Regression Coefficients for Face-to-Face vs. Telephone Modality*

|  | β | *p* |
| --- | --- | --- |
| **SEQ** |  |  |
| depth | -0.37 | .049 |
| smoothness | -0.04 | .842 |
| post-session positivity | 0.15 | .437 |
| **CSQ** |  |  |
| total score | -0.21 | .261 |
| **WAI-SR-P** |  |  |
| bond | -0.31 | .097 |
| tasks | -0.28 | .141 |
| goals | -0.03 | .882 |
| **WAI-SR-T** |  |  |
| bond | -0.27 | .128 |
| tasks | -0.31 | .089 |
| goals | -0.21 | .259 |

*Note*: Adjusted standardized beta coefficient for the predictor *modality* (0 = face-to-face, 1 = telephone). Adjustment was made for the variables *therapist* and *number of sessions*.
